# Enteric reabsorption processes and their impact on drug pharmacokinetics

**DOI:** 10.1038/s41598-021-85174-w

**Published:** 2021-03-11

**Authors:** Manuel Ibarra, Iñaki F. Trocóniz, Pietro Fagiolino

**Affiliations:** 1 Department of Pharmaceutical Sciences, Faculty of Chemistry, Universidad de la República, P.O. Box 1157, 11800 Montevideo, Uruguay; 2 Pharmacometrics and Systems Pharmacology Research Unit, Department of Pharmaceutical Technology and Chemistry, School of Pharmacy and Nutrition, University of Navarra, Pamplona, Spain; 3 IdiSNA, Navarra Institute for Health Research, Pamplona, Spain

**Keywords:** Clinical pharmacology, Pharmacokinetics, Pharmaceutics, Clinical pharmacology, Pharmacokinetics, Drug development

## Abstract

Enteric reabsorption occurs when a drug is secreted into the intestinal lumen and reabsorbed into the systemic circulation. This distribution process is evidenced by multiple peaks in pharmacokinetic profiles. Commonly, hepatobiliary drug secretion is assumed to be the underlying mechanism (enterohepatic reabsorption, EHR), neglecting other possible mechanisms such as gastric secretion (enterogastric reabsorption, EGR). In addition, the impact of drug reabsorption on systemic clearance, volume of distribution and bioavailability has been a subject of long-standing discussions. In this work, we propose semi-mechanistic pharmacokinetic models to reflect EHR and EGR and compare their respective impact on primary pharmacokinetic parameters. A simulation-based analysis was carried out considering three drug types with the potential for reabsorption, classified according to their primary route of elimination and their hepatic extraction: (A) hepatic metabolism—low extraction; (B) hepatic metabolism—intermediate/high extraction; (C) renal excretion. Results show that an increase in EHR can significantly reduce the clearance of drugs A and B, increase bioavailability of B drugs, and increase the volume of distribution for all drugs. Conversely, EGR had negligible impact in all pharmacokinetic parameters. Findings provide background to explain and forecast the role that this process can play in pharmacokinetic variability, including drug-drug interactions and disease states.

## Introduction

Drug reabsorption has always been a pharmacokinetic challenge, both in modeling blood/plasma multiple-peak drug profiles and in interpreting its impact on drug elimination clearance and bioavailability. Several extensive reviews with focus on enterohepatic reabsorption mechanistic aspects, modeling approaches and pharmacokinetic consequences have been published^1–4^. Interestingly, within these articles, the impact of drug reabsorption on fundamental pharmacokinetic parameters such as the systemic clearance and the oral bioavailability is undefined. On the other hand, it seems settled that both volume of distribution and drug half-life are increased with higher reabsorption.

Discussion about the impact of reabsorption in drug clearance and oral bioavailability has been around for a long time. In 1980, Veng-Pedersen and Miller developed a model to describe cimetidine plasma concentrations after single oral and intravenous doses^5^. The proposed model included a two-compartmental drug distribution structure plus a compartment accounting for the gallbladder, from where the drug was secreted into the gut in a discontinuous process. The liver was included as part of the central compartment and therefore, the first-pass effect was not mechanistically accounted for. Applying this model to cimetidine observations, authors suggested that the area under the plasma concentration–time curve (AUC) was dependent on the extent of reabsorbed drug, and since this process was dependent on the administration route the ratio between oral and intravenous AUC would not be a suitable measure of oral bioavailability. In reply to this work, Shepard et al.^6,7^, used the same and more complex models to deduce that drug clearance and oral bioavailability were independent of enterohepatic reabsorption and thus the AUC ratio was an unbiased estimator of the oral bioavailability. Although other authors have further argued with different methodologies that enterohepatic reabsorption increases AUC by decreasing systemic clearance^8,9^ and/or increasing oral bioavailability^8,10^, the current understanding of its pharmacokinetic impact remains unclear.

In this work, by using semi-mechanistic pharmacokinetic models, we analyze and discuss the impact of different mechanisms leading to enteric reabsorption on drug clearance, volume of distribution and bioavailability. In addition we aim to shed light into the long-standing discussion through a quantitative assessment of this impact, implementing the models in a middle-out approach to simulate pharmacokinetic outcomes for three drug groups defined according to disposition characteristics.

### Discerning between cycling/circulation and reabsorption

Enteric reabsorption occurs when a fraction of drug transferred from the arterial bloodstream to the gastrointestinal tract (GIT) is subsequently reabsorbed back into the systemic circulation. When this process includes a discrete step, it causes secondary or multiple peaks in the plasma-concentration–time profile. The most known process of this kind is usually referred to as enterohepatic cycling or enterohepatic circulation (EHC), however in this work we are making a distinction and suggesting the use of drug enterohepatic reabsorption (EHR) as a more precise concept. The cycling fraction of a compound undergoing EHC is the one that completes the cycle liver-gallbladder-gut-liver, diffusing through the intestinal epithelium in a significant extent, avoiding gut-wall mediated metabolism and resulting efficiently extracted by the liver. On the other hand, when EHR takes place, the drug reaches the systemic bloodstream after diffusing through the intestinal epithelium, avoiding the hepatic metabolism and hepatobiliary secretion, and completing the cycle bloodstream-liver-gallbladder-gut-bloodstream. Therefore, cycling is not a synonym of reabsorption. Bile acids are the compounds associated with the largest fraction undergoing EHC and are mostly confined to the hepatoportal system ^11^. It must be emphasized that drug secretion into the GIT does not necessarily lead to drug reabsorption, since drug in the GIT can also be excreted or metabolized. The outcome for intraluminal drug will depend on its physicochemical and biological characteristics.

### Mechanisms for drug enteric reabsorption

Enteric reabsorption is usually assumed to have an enterohepatic underlying mechanism, and many drugs and endogenous compounds are known to undergo EHR. Its characteristics and physiological details of the process have been discussed extensively elsewhere^1–3,12–14^.

Although less frequently reported, multiple-peak phenomena have been observed after intravenous administration of drugs with negligible bile secretion. As reviewed by Davies et al.^1^, enterogastric reabsorption (EGR), *i.e.* gastric secretion with subsequent intestinal reabsorption, has been observed for several basic drugs. Shore et al. reported in 1957 how intravenously administered basic drugs were secreted into the stomach in dogs, in a fraction that showed good correlation with drug pKa^15^. Other authors had similar findings for morphine and acetylmethadol as reviewed by Lynn et al. who reported a large methadone recovery in human gastric juice after parental injection^16^. EGR occurs after the drug is removed from the blood by parietal cells and secreted into the stomach lumen, a process conditioned by drug pKa, solubility and plasma unbound fraction. Extracted drug is therefore collected in the stomach until pylorus opening, which leads to the discharge of gastric content in the gut and to subsequent drug absorption.

## Methods

Two semi-mechanistic models accounting for key aspects of EHR and EGR were developed for pharmacokinetic assessment. Although different approaches can be implemented to address the questions raised here, this model-based approach allowed us to assess the phenomena under study in two ways, addressing the pharmacokinetic impact of drug enteric secretion and drug reabsorption.

First, to assess the impact of drug hepatobiliary and gastric secretion in primary pharmacokinetic parameters under models EHR and EGR respectively, theoretical equations were derived and verified against empirical estimations obtained through deterministic simulations.

Then, to quantitatively assess the impact of drug reabsorption on primary pharmacokinetic parameters, three drug types showing significant reabsorption were defined by assigning specific values to the first-order rate constants. Drugs undergoing reabsorption in a significant magnitude share the following characteristics to some extent: high enteric secretion (hepatobiliary, gastric, or other possible route), high intestinal permeability, and negligible intestinal elimination. Nevertheless, differences in distribution and elimination among these drugs could make the reabsorption process dissimilarly meaningful. These differences were evaluated by conducting simulations with variability in the first-order rate constants for each drug type in both models, estimating the primary pharmacokinetic parameters for the simulated concentrations by non-compartmental analysis and evaluating the correlation between these parameters and the magnitude of drug hepatobiliary (or gastric) secretion.

### Pharmacokinetic models

#### Enterohepatic reabsorption (EHR)

One key EHR feature that has not been taken explicitly into account by previous mathematical analyses^5–10^ relies on the interplay between hepatobiliary secretion and enzyme metabolism in the hepatocyte. Both endogenous and exogenous compounds are secreted into the bile by means of active transport, involving one of the several efflux transporters expressed at the hepatobiliary barrier. Along with hepatocyte drug uptake, efflux into the sinusoidal blood and into the bile will modify the amount of drug available for biotransformation, and although the interplay between influx/efflux transporters and metabolic enzymes at the liver and the gut-wall is now widely accepted, it has not been considered for evaluating the impact of reabsorption in drug pharmacokinetics. Among other authors, Kusuhara and Sugiyama addressed the impact of transporters on tissue selective drug distribution and elimination^17^, and highlighted the importance of rate-limiting uptake and efflux processes mediated by transporters in drug hepatic elimination^18^. Also in a previous publication, Fagiolino et al.^19^ showed how efflux activity at the hepatocyte could affect systemic elimination clearance. In this work, we apply these concepts into pharmacokinetic models with a focus on the analysis of enteric reabsorption.

**Figure 1:**
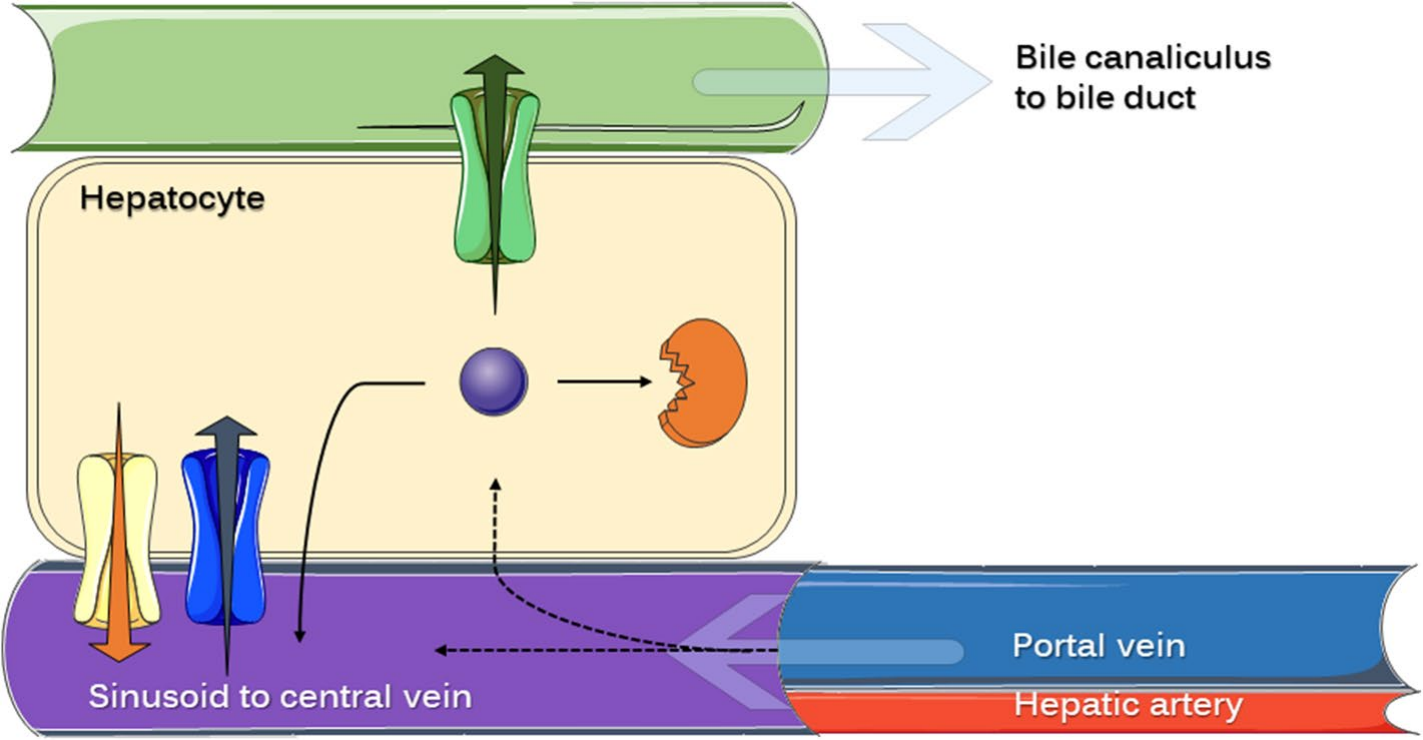
Schematic representation of the different routes that a drug can follow during its passage through the liver. The hepatocyte environment is shown, emphasizing the presence of drug transporters (influx and efflux) at the different membranes and the spatial relation with blood and bile circulation. Either arriving from the portal vein or the hepatic artery, drug circulating in liver sinusoids can be active or passively transferred into the hepatocyte or keep circulating towards the central vein (systemic circulation). Black dashed arrows represent these transferences. Intracellular drug (violet sphere) will be suffer one of these processes: hepatobiliary secretion (active efflux represented by green transporters), enzyme-mediated biotransformation (orange structure representing intracellular metabolic enzyme) or transference back into sinusoidal blood (active or passive). Yellow structures with orange arrow represent efflux transporters at the basolateral membrane (such as MRP3 and MRP4), while blue structures with blue arrow represent influx transporters (such as OATP). Bile canaliculus convey in the bile duct delivering the fluid into the gallbladder. This figure was prepared using content from Servier Medical Art, licensed under a Creative Common Attribution 3.0 Generic License. http://smart.servier.com/.

To fully understand the effect of the extent of EHR on drug clearance, volume of distribution and oral bioavailability through pharmacokinetic modeling, the competitive nature of the processes taking place in the hepatocyte must be conserved and quantitatively accounted for. Our view of this process is represented in Fig. 1, and comprises the following physiologically based principles:

- Drug reaching the liver from either portal vein or hepatic artery can be transported (active or passively) into the hepatocyte. A fraction of this incoming drug will continue its circulation through the sinusoidal blood reaching the central vein (systemic circulation), and thus avoiding the intracellular hepatocyte.
- Drug reaching the intracellular hepatocyte space will be subject to one of the following processes: (i) elimination by enzymatic metabolism, (ii) hepatobiliary secretion by active efflux into the bile canaliculus through the apical membrane, or (iii) transportation (active or passive) back into the sinusoids through the basolateral membrane. Of note: intracellular drug could eventually avoid hepatic extraction in this last process.

**Figure 2:**
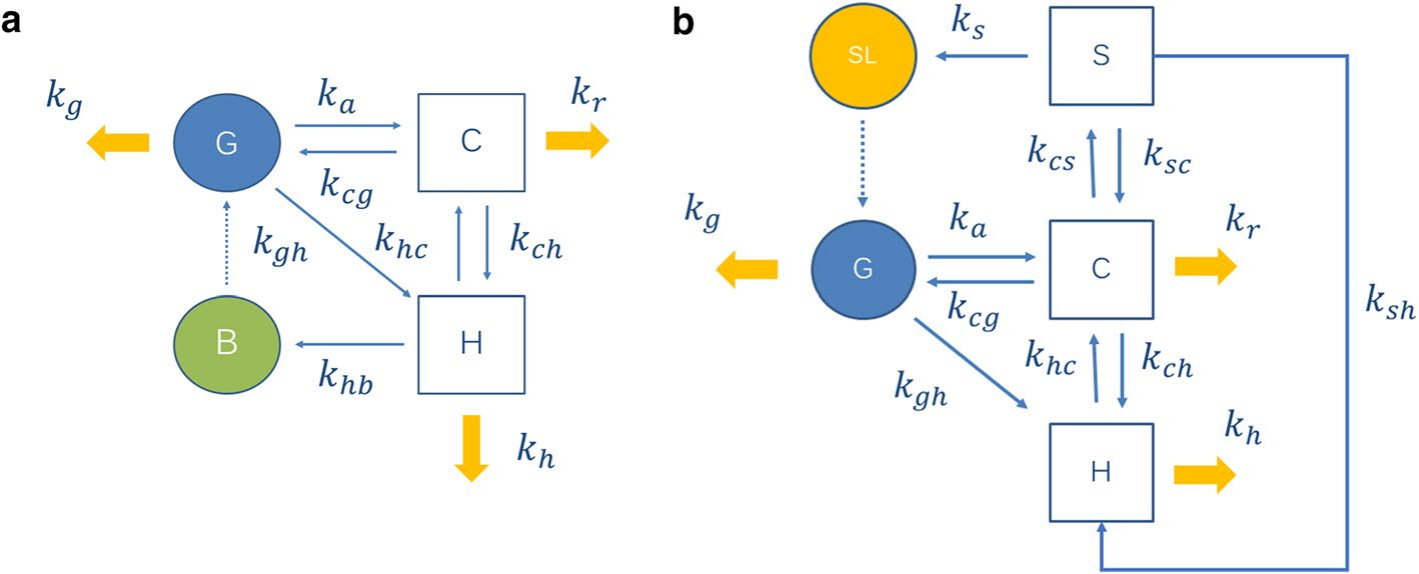
Pharmacokinetic model implemented in the analysis of (**a**) enterohepatic reabsorption (EHR), and (**b**) enterogastric reabsorption (EGR). Compartments: central compartment (C), gut (G), hepatocytes (H), gallbladder (B), stomach lumen (SL) and gastric parietal cells (S). First-order rate constants describing mass transferences: ${k}_{a}$ (drug absorption from the gut into the central compartment),${k}_{gh}$(hepatic uptake of drug coming from the gut through portal venous blood), ${k}_{g}$ (intestinal elimination), ${k}_{cg}$(drug transference from the systemic circulation to the gut), ${k}_{hc}$ (drug transference from the hepatocyte to the central compartment), ${k}_{ch}$ (drug transference from the central compartment to the hepatocyte),${k}_{h}$ (hepatic elimination), ${k}_{hb}$ (hepatobiliary secretion), ${k}_{cs}$ (drug transference from the central compartment to the parietal cells), ${k}_{sc}$ (drug transference from the parietal cells to the central compartment), ${k}_{s}$ (drug secretion from parietal cells into gastric lumen) ${k}_{sh}$ (hepatic uptake of drug coming from the parietal cells through the gastric vein), and ${k}_{r}$ (renal elimination).

These principles were implemented in the semi-mechanistic EHR model shown in Fig. 2a consisting in four compartments representing the gut (G), hepatocytes (H), gallbladder (B) and a central compartment (C) comprising all remaining body tissues and fluids, including the kidneys. The model explicitly represents three drug outputs from the central compartment: renal elimination, hepatocyte uptake and enterocyte (gut) uptake through the basolateral membrane. Three routes of systemic elimination are allowed: renal, hepatic, and intestinal (gut-wall mediated metabolism and intestinal excretion). Hepatobiliary secreted drug is partially and temporarily stored in the gallbladder, and subsequently delivered into the gut in a time-varying process *b(t)* including continuous and discrete secretions.

Importantly, the representation of the hepatocyte as a separated compartment is key to mathematically acknowledge the competition between the output routes described before.

In the case of the gastrointestinal organs, the lumen and the gut-wall are lumped in a unique compartment since focus is not directed to kinetic competition in the intestine. From this compartment, the drug can be absorbed into the bloodstream, extracted into the hepatocytes or result eliminated by either fecal excretion or gut-wall mediated metabolism. Intestinal-mediated elimination often has a minor contribution to systemic clearance, mainly because of the reduced basolateral drug input to the enterocytes and the lower enzyme density in gut in relation to liver. However, for drugs efficiently distributed into the gut wall during its passage through the mesenteric arteries and ramifications, which in turn are substrates of enzymes present at the gut (e.g. CYP3A4), this process should not be neglected.

The following differential equations represent the time dependent drug amount variation in the different compartments:

1 $$\frac{{dA}_{C}}{dt}= {k}_{a}{A}_{G}+ {k}_{hc}{A}_{H}-\left({k}_{ch}+{{k}_{cg}+k}_{r}\right){A}_{C}$$

2 $$\frac{{dA}_{H}}{dt}= {k}_{ch}{A}_{C}+ {k}_{gh}{A}_{G}-\left({k}_{hc}+{{k}_{hb}+k}_{h}\right){A}_{H}$$

3 $$\frac{{dA}_{B}}{dt}= {k}_{hb}{A}_{H}-b(t)$$

4 $$\frac{{dA}_{G}}{dt}= {k}_{cg}{A}_{C}+ b(t)-\left({k}_{a}+{{k}_{gh}+k}_{g}\right){A}_{G}$$

where ${A}_{C}$, ${A}_{H}$, ${A}_{B}$ and ${A}_{G}$ stands for drug amount in the central, hepatocyte, gallbladder, and intestinal compartments, respectively. First-order rate constants for mass transferences are ${k}_{a}$ (drug absorption from the gut into the central compartment), ${k}_{hc}$ (drug transference from the hepatocyte to the central compartment), ${k}_{ch}$ (drug transference from the central compartment to the hepatocyte), ${k}_{cg}$(drug transference from the systemic circulation to the gut), ${k}_{gh}$(hepatic uptake of drug coming from the gut through portal venous blood), ${k}_{hb}$ (hepatobiliary secretion), ${k}_{r}$ (renal elimination), ${k}_{h}$ (hepatic elimination) and ${k}_{g}$ (intestinal elimination). Of note, ${k}_{a}$ reflects intestinal drug absorption bypassing the hepatocytes throughout liver capillaries and extracellular space.

#### Enterogastric reabsorption (EGR)

To represent EGR (Fig. 2b), the same principles described above were implemented. The stomach was separated from the gastrointestinal space and displayed in two separated compartments: the gastric lumen (SL) and the gastric parietal cells (S). Inclusion of the latter compartment in the model intended to reflect the blood supply and drug transference with a semi-mechanistic approach. Hence, compounds reaching the parietal cells from the bloodstream through gastric and gastroduodenal arteries can be thereafter secreted into the gastric lumen, or continue via portal blood towards the liver, where a fraction can be extracted and the rest will go back to the systemic circulation. Drug secretion into the gastric lumen is represented by the first-order rate constant ${k}_{s}$. In the stomach, the drug can be partially and temporarily stored, previous to be transferred to the gut lumen in a variable process involving continuous and discrete transferences, jointly described with *s(t)*. The following differential equations describe the drug mass balance within the model:

5 $$\frac{{dA}_{C}}{dt}= {k}_{a}{A}_{G}+ {k}_{hc}{A}_{H}-\left({k}_{ch}+{{k}_{cg}+{k}_{cs}+k}_{r}\right){A}_{C}$$

6 $$\frac{{dA}_{H}}{dt}= {k}_{ch}{A}_{C}+ {k}_{gh}{A}_{G}+{k}_{sh}{A}_{S}-\left({k}_{hc}{+k}_{h}\right){A}_{H}$$

7 $$\frac{{dA}_{S}}{dt}= {k}_{cs}{A}_{C}-\left({k}_{sc}+{{k}_{sh}+k}_{s}\right){A}_{S}$$

8 $$\frac{{dA}_{SL}}{dt}= {k}_{s}{A}_{S}- s(t)$$

9 $$\frac{{dA}_{G}}{dt}= {k}_{cg}{A}_{C}+ s(t)-\left({k}_{a}+{{k}_{gh}+k}_{g}\right){A}_{G}$$

With ${A}_{C}$, ${A}_{H}$, ${A}_{S}$, ${A}_{SL}$ and ${A}_{G}$ standing for the amount of drug in the central, hepatocyte, parietal cell, stomach lumen and gut compartment, respectively. At the parietal cells compartment, drug input is governed by first-order rate constant ${k}_{cs}$, while ${k}_{sc}$, ${k}_{s}$ and ${k}_{sh}$ are first-order rate constants that determine the output to the central compartment, the gastric lumen, and the hepatocytes, respectively.

##### *Assumptions*

The semi-mechanistic pharmacokinetic models proposed were developed to analyze the impact of drug reabsorption in the systemic and temporal exposure of the body to different compounds. Several assumptions were made to simplify the analysis: (i) all drug transferences are considered to follow first-order kinetics, (ii) with exception of the gastrointestinal tract, gallbladder and liver, all organs are lumped into the central compartment, assuming that drug concentrations in these tissues and fluids reach an instantaneous kinetic equilibrium; (iii) drug elimination beyond hepatic, renal and intestinal is negligible; (iv) the parent drug is the secreted compound: the process of drug reabsorption implying biotransformation, metabolite enteric secretion, back-conversion to the parent drug in the gut and subsequent absorption is simplified in two steps, which are enteric secretion and parent drug absorption; and (v) in the EHR model, drug absorption through the gastric mucosa as well as the transference to parietal cells are neglected processes. Similarly, in the EGR model, drug hepatobiliary secretion is neglected.

### Derivation of theoretical equations

#### Systemic clearance under the proposed models

A theoretical equation for the systemic clearance (CL), defined as the elimination clearance measured from the central compartment, was derived for each model in order to visualize the impact of hepatobiliary and gastric secretions. The method described by Shepard et al.^6^ was implemented to obtain an equation for the area under the curve for the concentration of drug in central compartment over the time interval *t* = *0* to *t* = *∞* (AUC_1_) after a single bolus administration of drug into the same compartment (dose = D). The equation for CL is then obtained as $CL = {D /AUC_{1} }$. The implemented symbols are shown in Table 1.

**Table 1:** Symbols implemented for deriving theoretical equations of pharmacokinetic parameters.

Symbol	Equation	Description
**Symbols corresponding to the EHR model**		
$\Omega$	$\Omega = {k}_{hc}+{{k}_{hb}+k}_{h}$	First-order rate constant accounting for total drug output from the hepatocyte
$\Phi$	$\Phi = {k}_{a}+{{k}_{gh}+k}_{g}$	First-order rate constant accounting for total drug output from the gut
${F}_{a}$	${F}_{a}= {k}_{a}/\left({k}_{a}+{{k}_{gh}+k}_{g}\right)$	Fraction of drug being transferred from the gut to the central compartment
${E}_{h}$	${E}_{h}= {k}_{h}/\left({k}_{hc}+{{k}_{hb}+k}_{h}\right)$	Fraction of intra-hepatocytic drug submitted to irreversible metabolism
${E}_{gh}$	${E}_{gh}= {k}_{gh}/\left({k}_{a}+{{k}_{gh}+k}_{g}\right)$	Fraction of drug being transferred from the gut to the hepatocyte
${E}_{hb}$	${E}_{hb}= {k}_{hb}/\left({k}_{hc}+{{k}_{hb}+k}_{h}\right)$	Fraction of intra-hepatocytic drug submitted to hepatobiliary secretion
${E}_{hc}$	${E}_{hc}= {k}_{hc}/\left({k}_{hc}+{{k}_{hb}+k}_{h}\right)$	Fraction of drug being transferred from the hepatocyte to the central compartment
**Symbols corresponding to the EGR model**		
${\Omega }^{^{\prime}}$	${\Omega }^{^{\prime}}= {k}_{hc}{+k}_{h}$	First-order rate constant accounting for total drug output from the hepatocyte
$\Phi$	$\Phi = {k}_{a}+{{k}_{gh}+k}_{g}$	First-order rate constant accounting for total drug output from the gut
$\Theta$	$\Theta = {k}_{sc}+{{k}_{sh}+k}_{s}$	First-order rate constant accounting for total drug output from the parietal cells compartment
${E}_{h}$	${E}_{h}= {k}_{h}/\left({k}_{hc}{+k}_{h}\right)$	Fraction of intra-hepatocytic drug submitted to irreversible metabolism
${E}_{s}$	${E}_{s}={k}_{s}/({k}_{s}+{k}_{sh}+{k}_{sc})$	Fraction of drug in the parietal cells being submitted to gastric secretion

First-order rate constants describing mass transferences: ${k}_{a}$ (drug absorption from the gut into the central compartment),${k}_{gh}$(hepatic uptake of drug coming from the gut through portal venous blood), ${k}_{g}$ (intestinal elimination), ${k}_{cg}$(drug transference from the systemic circulation to the gut), ${k}_{hc}$ (drug transference from the hepatocyte to the central compartment), ${k}_{ch}$ (drug transference from the central compartment to the hepatocyte),${k}_{h}$ (hepatic elimination), ${k}_{hb}$ (hepatobiliary secretion), ${k}_{cs}$ (drug transference from the central compartment to the parietal cells), ${k}_{sc}$ (drug transference from the parietal cells to the central compartment), ${k}_{s}$ (drug secretion from parietal cells into gastric lumen) and ${k}_{sh}$ (hepatic uptake of drug coming from the parietal cells through the gastric vein).

#### Volume of distribution under the proposed models

When drug reabsorption occurs, the discrete nature of the process makes the volume of distribution (Vd), defined as the total amount of drug in the system ($${A}_{T})$$ over drug concentration in the central compartment (Cc), to change with time, even under the steady state of an intravenous perfusion with constant drug input rate. In this situation, Vd follows an oscillating pattern, decreasing after drug reabsorption and increasing to a maximum value right before gallbladder/gastric release. In order to deduce an equation for a mean Vss ($$\stackrel{-}{{V}_{SS}}$$), drug transference from the gallbladder and the stomach lumen into the gut was simplified to a continuous process following first-order kinetics, introducing therefore a slight change in both models, which will be referred to as EHR’ and EGR’. These models were analyzed in a steady state condition for an intravenous infusion at a constant rate Ro, where drug amount is constant in all compartments and therefore $$\frac{{dA}_{x}}{dt}=0$$. A theoretical equation for $$\stackrel{-}{{V}_{SS}}$$ was obtained after reaching an expression for the total drug amount in the system of n compartments ($${A}_{Tss}=\sum_{i=1}^{i=n}{A}_{i}$$ , being $$i$$ the compartment number) and implementing the definition $$ \overline{{V_{{SS}} }}  = {{A_{{TSS}} } \mathord{\left/ {\vphantom {{A_{{TSS}} } {C_{c} }}} \right. \kern-\nulldelimiterspace} {C_{c} }} $$. The implemented symbols are shown in Table 1.

#### Oral bioavailability under the EHR model

No impact of drug reabsorption in oral bioavailability is expected under the EGR model given that here the drug secreted into the gastric lumen and subsequently reabsorbed comes originally from the central compartment and therefore it is already part of the bioavailable fraction. Conversely, when enterohepatic reabsorption is present, a fraction of dose passing through the liver after an oral administration can be extracted into the hepatocytes and secreted back into the gut, having a subsequent opportunity for reaching the bloodstream and become bioavailable. To assess the impact of drug reabsorption on the dose fraction reaching the systemic circulation after an oral administration, a similar deduction to that developed by Peris-Ribera et al. was implemented for the EHR model^8^. The scheme shown in Table 2 explains how the partial fractions of drug reaching the central compartment after an oral administration (bolus into the gastrointestinal compartment) can be accounted for, while implemented symbols are included in Table 1.

**Table 2:**
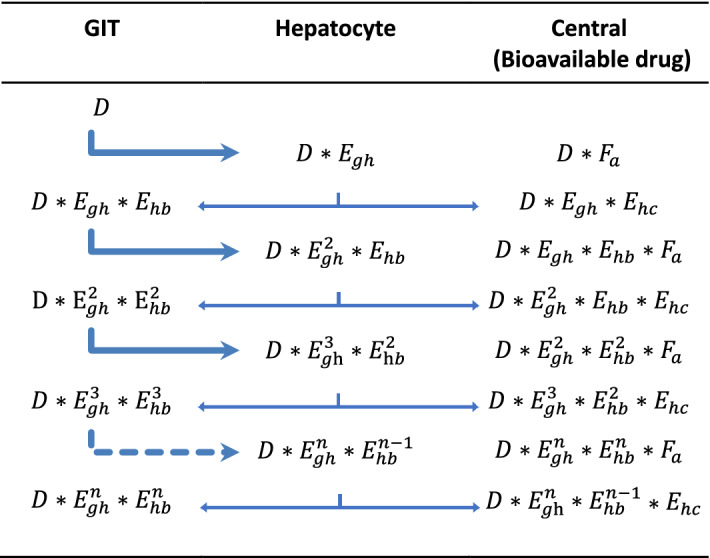
Fractional method for arriving to an equation for oral drug bioavailability under enterohepatic reabsorption (EHR model).

$D$: oral dose; ${E}_{gh}$: hepatocyte extraction ratio; ${F}_{a}:$ fraction of drug that reaches the systemic circulation from the gut; ${E}_{hb}:$ fraction of intra-hepatocytic drug that is submitted to hepatobiliary secretion; ${E}_{hc}: \text{fraction of intra-hepatocytic drug that reaches the systemic circulation.}$ See Table 1 for a mathematical description of these fractions.

### Model implementation and verification of derived model dependent equations for PK parameters

Both models were implemented through Mlxtran language in the R environment version 3.5.1 ^20^ using the *mlxR* package ^21^, a constitutive tool of the Monolix Suite 2018 ^22^ (Lixoft, France). Concentration versus time profiles for different situations were simulated with the *simulx* function with a time step of 0.1 h, and pharmacokinetic metrics such as the area under the simulated concentration–time profile from zero to infinity (AUC) were computed by non-compartmental analysis using the *exposure* function. Hence, the expressions derived for the relevant pharmacokinetic parameters shown in the results section were verified using model independent equations as follows.

For verification of CL and F equations, two administrations were simulated: a bolus dose (D) into the central (IV bolus) and into the gastrointestinal compartment (PO). The AUC was computed for each administration route, assuming infinity as a time higher than 7 empirical elimination half-lives. The systemic clearance was estimated as D/AUC_IV_, while partial empirical clearances (renal, hepatic, and intestinal) were computed as ΔE_x_/AUC_IV_, being ΔE_x_ the drug amount eliminated through the respective route, obtained from the simulations by integration of $\frac{\Delta {E}_{x}}{dt}$. Bioavailability was estimated as AUC_PO_/AUC_IV_. For computation of the volume of distribution at steady state, an IV perfusion at a constant rate Ro was simulated in models EHR’ and EGR’ until steady state. Under this condition, the empirical volume of distribution at steady state was computed dividing the sum of drug amounts in each compartment over the concentration in the central compartment. The equations were verified when the difference between the theoretical and empirical result was below 0.1%. This procedure was followed for all types of drugs defined in results section.

### Simulations assessing the impact of drug reabsorption

To evaluate the impact of drug reabsorption in the pharmacokinetic parameters of interest under the EHR and EGR models respectively, simulations (N = 1000) with variability on the first-order rate constants were performed considering different scenarios as follows. Different routes of administration were simulated: IV bolus to assess the impact on CL, IV perfusion to assess the impact on Vss (using models EHR’ and EGR’), and PO administration to assess the impact on F in the EHR model. In terms of coefficient of variation, the included variability was 30% in the absorption first-order rate constant (${k}_{a}$), 20% in elimination first-order rate constants (${k}_{h}$, ${k}_{g}$ and ${k}_{r}$) and 10% in other distribution first-order rate constants. For first-order rate constants ${k}_{hb}$ and ${k}_{s}$, a variability of 100% was included. The analysis was divided in sets of mean values for the first-order rate constants. The first criterion was to include drugs where hepatobiliary or gastric secretion leads to drug reabsorption. These drugs were then divided in three groups according to two factors that can have a major implication in the outcome: the elimination route and the hepatic extraction ${E}_{h}$. Refer to Table 1 for the mathematical definition of ${E}_{h}$ under each model. Example drugs included in each category were reported to display multiple peaks attributable to enteric reabsorption in humans.

A. Hepatic metabolism as the primary elimination route, with low hepatic extraction (${E}_{h}$~30%). Renal and intestinal elimination as secondary routes. Example drugs: amiodarone^2,3^, azithromycin^2,12^, ezetimibe^2,3,12^, lorazepam^2,3,12^, meloxicam^2,12^, methadone^12,16^, methotrexate^2,3^, mycophenolic acid^2,3^, nevirapine^23,24^, phenytoin^3,12,25^, rifampicin^2,3,12^, valproic acid^3,12,26^, warfarin^2,3^.
B. Hepatic metabolism as the primary elimination route, with intermediate/high hepatic extraction (${E}_{h}$>50%). Renal and intestinal elimination as secondary routes. Example drugs: chloramphenicol^2,3^, desipramine^27^, diltiazem^12^, fimasartan^28^, morphine^2,3^, naloxegol^12^, propranolol^29^, verapamil^30^.
C. Renal excretion as the primary elimination route. Hepatic metabolism as secondary route, with low hepatic extraction (${E}_{h}$<30%). Example drugs: ceftriaxone^2,3^, cimetidine^5,12^, doxycycline^2,3^, etintidine^12^, digoxin^3,12^, oseltamivir carboxylate^12^.

Drugs with high intestinal elimination were not considered given that its enteric secretion would be followed by intestinal elimination instead of drug reabsorption. Similarly, reabsorption is limited for drugs with high hepatic extraction. The magnitudes assigned to each model parameter were selected to represent mean drugs within each class. Table 3 summarizes the mean pharmacokinetic parameters of each drug type. Scripts and specific values given to all first-order rate constants are supplied as supplementary information.

**Table 3:** Pharmacokinetic characteristics defined for each drug type to conduct the sensitivity analysis of drug reabsorption in EHR and EGR models.

Drug	${{\varvec{C}}{\varvec{L}}}_{{\varvec{h}}}\boldsymbol{ }({\varvec{L}}/{\varvec{h}})$	${{\varvec{C}}{\varvec{L}}}_{{\varvec{g}}}\boldsymbol{ }({\varvec{L}}/{\varvec{h}})$	${{\varvec{C}}{\varvec{L}}}_{{\varvec{r}}}\boldsymbol{ }({\varvec{L}}/{\varvec{h}})$	${{\varvec{V}}}_{{\varvec{s}}{\varvec{s}}}\boldsymbol{ }({\varvec{L}})$	${\varvec{F}}$	${{\varvec{E}}}_{{\varvec{h}}{\varvec{b}}}$
**Mean parameters for the EHR model**						
A	0.79	0.093	0.10	12	0.94	0.22
B	7.2	0.91	0.90	13	0.58	0.26
C	1.0	0.0	4.0	15	0.98	0.26

Drug	${{\varvec{C}}{\varvec{L}}}_{{\varvec{h}}}\boldsymbol{ }({\varvec{L}}/{\varvec{h}})$	${{\varvec{C}}{\varvec{L}}}_{{\varvec{g}}}\boldsymbol{ }({\varvec{L}}/{\varvec{h}})$	${{\varvec{C}}{\varvec{L}}}_{{\varvec{r}}}\boldsymbol{ }({\varvec{L}}/{\varvec{h}})$	${{\varvec{V}}}_{{\varvec{s}}{\varvec{s}}}\boldsymbol{ }({\varvec{L}})$	${\varvec{F}}$	${{\varvec{E}}}_{{\varvec{s}}}$
**Mean parameters for the EGR model**						
A	3.0	0.32	0.30	16.4	0.84	0.45
B	8.6	0.43	1.5	14.3	0.58	0.45
C	1.1	0.0	4.0	17.3	0.98	0.45

${CL}_{h}$: hepatic clearance; ${CL}_{g}$: intestinal clearance, including excretion and gut-wall mediated metabolism; ${CL}_{r}:$ renal clearance; ${V}_{ss}$: volume of distribution at steady state; $F$: oral bioavailability; ${E}_{hb}$: fraction of intra-hepatocytic drug that is submitted to hepatobiliary secretion;${E}_{s}$: fraction of drug extracted to the stomach lumen from the parietal cells.

#### *Assumptions*

In all cases, transference across intestinal epithelium was not considered a limitation for drug absorption and intestinal elimination was assumed to have a minor contribution to systemic clearance. This criterion is followed for simplicity, to include in the analysis drugs that can have a significant enteric reabsorption and exclude drugs for which hepatobiliary and gastric secretion will lead to drug excretion increasing elimination clearance.

Gallbladder and gastric emptying in EHR and EGR models respectively were regarded as discrete and instantaneous events following meal intakes assumed to take place every 8 h, simulating therefore three diary reabsorption events. The basal secretion for both processes was neglected.

The mean ${k}_{hb}$ value in the EHR model corresponded to a ${E}_{hb}$ of ~ 25%. In the case of gastric drug secretion, the mean ${k}_{s}$ was defined to give a mean extraction into the stomach lumen (${E}_{s}$) of ~ 40%, being ${E}_{s}={k}_{s}/({k}_{s}+{k}_{sh}+{k}_{sc})$. These mean extraction values were defined accorded to our previous findings for valproic acid ^26^ and nevirapine ^23^, which were regarded as suitable to cover a wide range of extraction fractions in the simulations where a high variability was included for ${k}_{hb}$ and ${k}_{s}$.

Finally, no correlations were considered between random effects of the first-order rate constants, i.e. all drug transferences were assumed to vary independently. The focus of these simulations stands on the impact of a change in the magnitude of the enteric secretion (hepatobiliary or gastric) in pharmacokinetic parameters. This effect comes as a result of the competition between processes at the hepatocyte in the EHR model and at the parietal cells in the EGR model. A change in the hepatobiliary secretion of a compound is expected after an induction/inhibition of the active transport at the hepatobiliary barrier, which does not necessarily have to affect the other drug outputs from the hepatocyte (biotransformation and transference into the systemic circulation). Also, when comparing characteristics of different drugs, the rate of an active process like hepatobiliary secretion could be independent from the other outputs. In the case of gastric secretion, its rate could change following a change in gastric fluid secretions and intragastric pH, which would not affect other drug transferences. If different drugs are compared, the pKa could be a determinant on the magnitude of the secretion. Although this factor could affect other drug transferences within the body, for simplicity this effect was not considered.

## Results

### Equations for systemic clearance under the proposed models

#### Enterohepatic reabsorption (EHR model)

The total CL can be mathematically described by the following equation:

10 $$CL= {V}_{C}*\left[{k}_{r}+\frac{{k}_{h}\left({k}_{ch}({k}_{a}+{k}_{gh})+{{k}_{cg}k}_{gh}\right)+{k}_{g}\left({k}_{cg}({k}_{hc}+{k}_{hb})+{{k}_{ch}k}_{hb}\right)+{k}_{h}{k}_{g}({k}_{ch}+{k}_{cg})}{\left({k}_{h}+{k}_{hc}\right)\Phi +{k}_{hb}\left({k}_{a}+{k}_{g}\right)}\right]$$

where $\Phi$ stands for the first-order rate constant accounting for total drug output from the gut. It can be observed that the renal contribution to systemic CL (${CL}_{r}= {k}_{r}*{V}_{C}$ ) is independent from other routes of elimination, i.e. it can be estimated with the same equation no matter how significant the gut-wall and the hepatic clearances are. Conversely, the contributions of the latter two routes are correlated to some extent, shown by the term: ${k}_{h}{k}_{g}({k}_{ch}+{k}_{cg})$, in the numerator of Eq. (10). This happens for routes of elimination taking place from a peripheric compartment when a mass transference exists between the elimination compartments. If we assume a null contribution of the gut-wall metabolism and intestinal excretion to systemic CL, we reach an equation for the hepatic clearance:

11 $${CL}_{h}= {V}_{C}*\left[\frac{{k}_{h}\left({k}_{ch}\left({k}_{a}+{k}_{gh}\right)+{{k}_{cg}k}_{gh}\right)}{\left({k}_{h}+{k}_{hc}\right)\left({k}_{a}+{k}_{gh}\right)+{k}_{hb}{k}_{a}}\right]$$

This could be case for drugs which (i) are not substrates of enzymes expressed at the gut-wall, and (ii) are efficiently absorbed at the gut-wall, therefore showing insignificant intestinal excretion.

If, however, the hepatic clearance is negligible, intestinal contribution to systemic clearance can be estimated as:

12 $${CL}_{g}= {V}_{C}*\left[\frac{{k}_{g}\left({k}_{cg}\left({k}_{hc}+{k}_{hb}\right)+{{k}_{ch}k}_{hb}\right)}{{k}_{hc}\Phi +{k}_{hb}\left({k}_{a}+{k}_{g}\right)}\right]$$

#### Enterogastric reabsorption (EGR model)

Working with this model, the following equations are derived:

13 $${CL}_{r}= {V}_{C}*{k}_{r}$$

14 $${CL}_{h}= {V}_{C}*\left[\frac{{k}_{h}}{{\Omega }^{^{\prime}}}\left({k}_{ch}+\frac{{k}_{cs}({k}_{sh}\Phi +{k}_{s}{k}_{gh})}{\mathrm{\Theta \Phi }}+\frac{{k}_{cg}{k}_{gh}}{\Phi }\right)\right]$$

15 $${CL}_{g}= {V}_{C}*\left[\frac{{k}_{g}}{\Phi }\left({k}_{cg}+\frac{{k}_{cs}{k}_{s}}{\Theta }\right)\right]$$

where ${\Omega }^{^{\prime}},\Phi$ and $\Theta$ stands for the first-order rate constants accounting for total drug output from the hepatocyte, the gut, and the parietal cells compartment, respectively. In this model, hepatic and intestinal clearances are independent since there is no drug transference from the liver to the gut lumen. The total clearance can be obtained in any situation by summing the clearances of each elimination route.

### Equations for the volume of distribution under the proposed models

#### Enterohepatic reabsorption (EHR model)

Proceeding as explained, the following equation for the mean volume of distribution at steady state was derived:

16 $$\stackrel{-}{{V}_{SS}}= {V}_{C}*\left[1+\frac{{k}_{cg}}{\Phi }+\frac{{k}_{ch}\Phi +{k}_{gh}{k}_{cg}}{\mathrm{\Phi \Omega }-{k}_{hb}{k}_{gh}}*(1+\frac{{k}_{hb}}{\Phi }+\frac{{k}_{hb}}{{k}_{bg}})\right]$$

Here, $\Omega$ and $\Phi$ are first-order rate constants accounting for total drug output from the hepatocyte and gut respectively, while ${k}_{bg}$ stands for the first-order rate constant of drug transference from the gallbladder to the gut lumen. Its effect on ${V}_{SS}$ is evident: when the transference is slower, the drug retained in the gallbladder at steady state is higher and therefore the ${V}_{SS}$ result increased.

#### Enterogastric reabsorption (EGR model)

Following a similar procedure, the volume of distribution at steady state for the case where gastric drug secretion is present can be estimated as:

17 $$\stackrel{-}{{V}_{SS}}= {V}_{C}*\left[1+\frac{{k}_{ch}}{{\Omega }^{^{\prime}}}+\frac{{k}_{cs}}{\Theta }*\left(1+\frac{{k}_{sh}}{{\Omega }^{^{\prime}}}\right)+\frac{{k}_{s}{k}_{cs}+{k}_{cg}\Theta }{\mathrm{\Phi \Theta }}*\left(1+\frac{{k}_{gh}}{{\Omega }^{^{\prime}}}\right)+\frac{{k}_{s}{k}_{cs}}{{k}_{sg}\Theta }\right]$$

Here, ${k}_{sg}$ stands for the first-order rate constant of drug transference from the stomach to the gut lumen. Its effect on $\stackrel{-}{{V}_{SS}}$ is analogous to the gallbladder release in the EHR model, when the transference is slower, the drug retained in the stomach at steady state is higher and therefore the $\stackrel{-}{{V}_{SS}}$ result increased.

### Equation for drug bioavailability under enterohepatic reabsorption

An equation for the oral bioavailability (F) can be obtained, considering all drug fractions entering the central compartment from the GIT. The total amount of bioavailable drug can be obtained by summing all terms included under the third column of Table 2:

18 $$ \begin{aligned} D*F & = D*F_{a} *\left( {1 + E_{gh} E_{hb} + \left( {E_{gh} E_{hb} } \right)^{2} + \left( {E_{gh} E_{hb} } \right)^{3} + \cdots + \left( {E_{gh} E_{hb} } \right)^{n} } \right) \\ & \quad + D*E_{gh} *E_{hc} *\left( {1 + E_{gh} E_{hb} + \left( {E_{gh} E_{hb} } \right)^{2} + \left( {E_{gh} E_{hb} } \right)^{3} + \ldots + \left( {E_{gh} E_{hb} } \right)^{n} } \right) \\ \end{aligned} $$

where D represents the given dose, ${F}_{a}$ the fraction of drug being transferred from the gut to the central compartment, ${E}_{gh}$ the fraction of drug being transferred from the gut to the hepatocyte, ${E}_{hb}$ the fraction of intra-hepatocytic drug submitted to hepatobiliary secretion and ${E}_{hc}$ the fraction of drug being transferred from the hepatocyte to the central compartment. The term inside the brackets can be further reduced by multiplying the numerator and denominator by ($1-{E}_{gh}{E}_{hb}$):

19 $$\left(1+{E}_{gh}{E}_{hb}+{\left({E}_{gh}{E}_{hb}\right)}^{2}+{\left({E}_{gh}{E}_{hb}\right)}^{3}+\dots +{\left({E}_{gh}{E}_{hb}\right)}^{n}\right)*\frac{\left(1-{E}_{gh}{E}_{hb}\right)}{\left(1-{E}_{gh}{E}_{hb}\right)}=\frac{1}{\left(1-{E}_{gh}{E}_{hb}\right)}$$

Then, an equation for F is obtained:

20 $$F= \frac{{F}_{a}+{E}_{gh}{E}_{hc}}{(1-{E}_{gh}{E}_{hb})}$$

The impact of drug hepatobiliary secretion on F is therefore dependent on drug extraction into the hepatocyte during its passage through the hepatic portal circulation.

### Simulations assessing the impact of drug reabsorption

**Figure 3:**
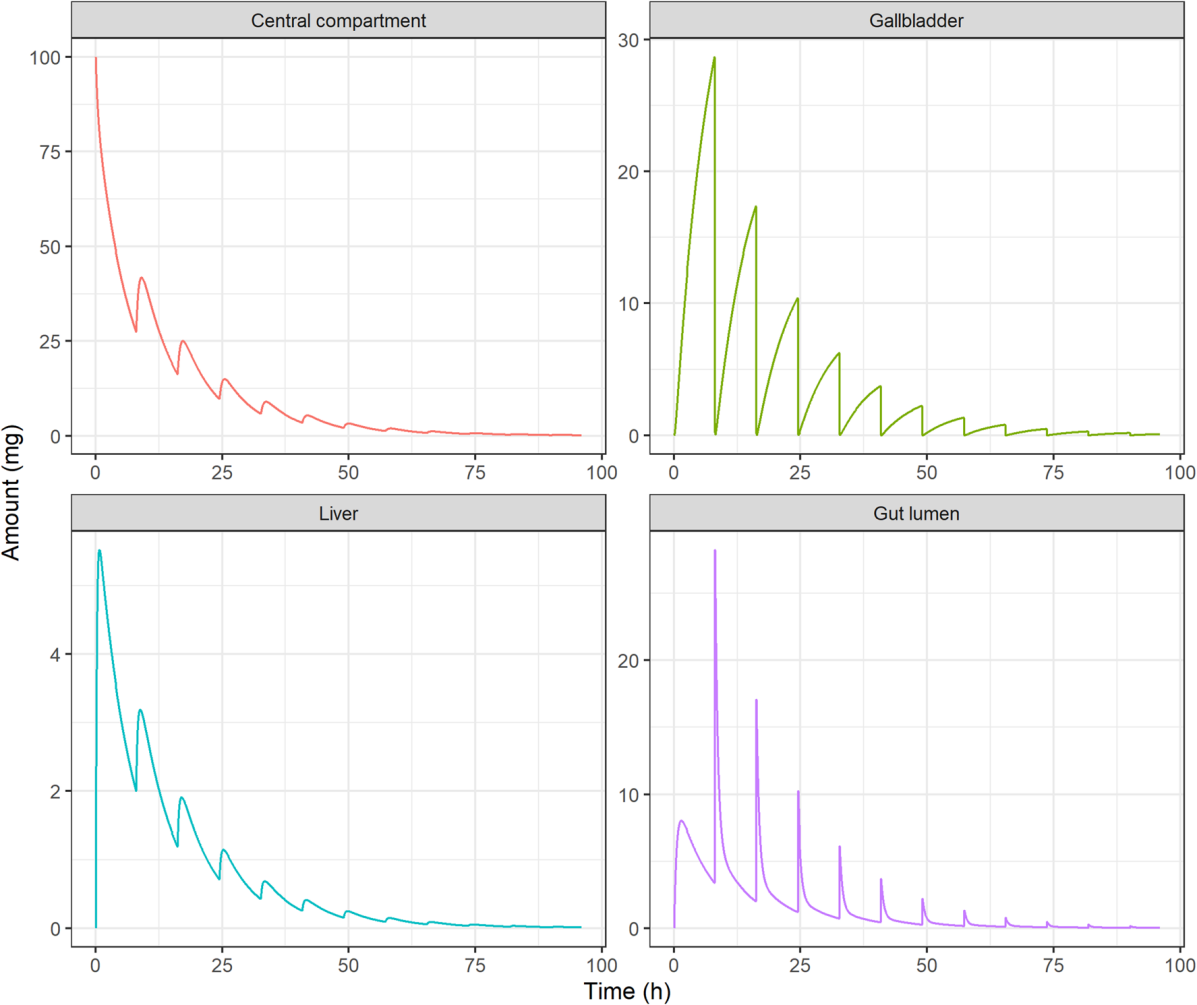
Pharmacokinetic profiles simulated for an intravenous administration of 100 mg, drug A, in the EHR model. Drug amounts in the central compartment (${A}_{C}$), gallbladder (${A}_{B}$), liver (${A}_{H}$) and gut lumen (${A}_{G}$) are plotted versus time after dose. The multiple-peaking nature of the profiles corresponds to discrete gallbladder emptying events taking place every 8 h after meal intake and followed by drug reabsorption into the central compartment.

**Figure 4:**
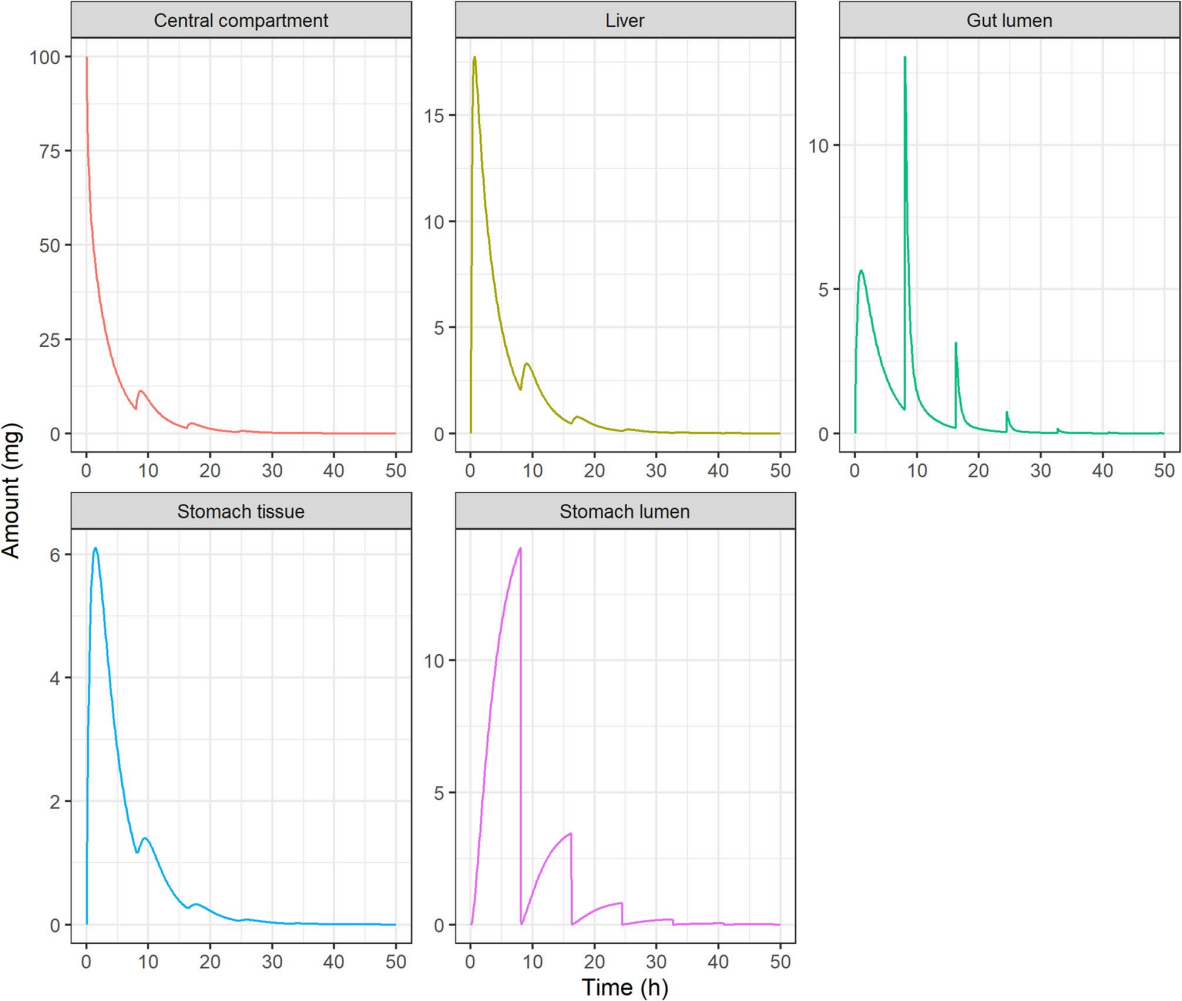
Pharmacokinetic profiles simulated for an intravenous administration of 100 mg, drug A, in the EGR model. Drug amounts in the central compartment (${A}_{C}$), stomach tissue (${A}_{S}$), liver (${A}_{H}$), gut lumen (${A}_{G}$) and stomach lumen (${A}_{SL}$) are plotted versus time after dose. The multiple-peaking nature of the profiles corresponds to discrete gastric emptying events taking place every 8 h after with meal intake and followed by drug reabsorption into the central compartment.

Figures 3 and 4 show deterministic simulations for the IV bolus administration in the EHR and EGR models respectively for drug A.

**Figure 5:**
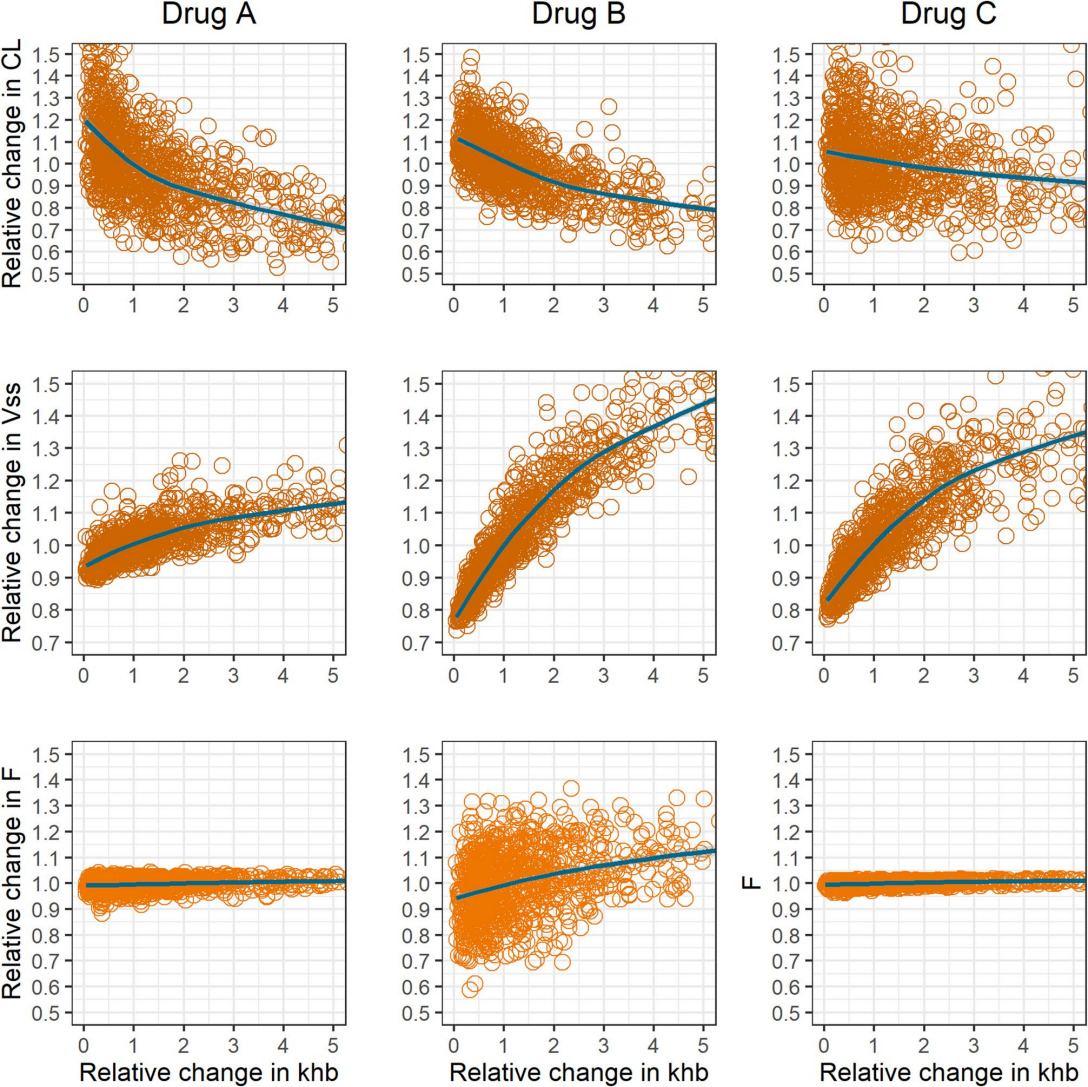
Sensitivity analysis with variability in all first-order rate constants, shown as scatterplots, for the mean impact of drug hepatobiliary secretion first-order rate constant (${k}_{hb}$) on systemic clearance (CL), volume of distribution at steady state (Vss) and oral bioavailability (F).

Figure 5 shows the correlation between the relative change in the empirical systemic CL, ${V}_{SS}$ and F versus the relative change in the magnitude of ${k}_{hb}$ obtained with the EHR model for the different drug types defined. It can be observed that an increase in ${k}_{hb}$ (i.e. increased rate constant defining the movement of drug from hepatocyte into the bile) produces a significant decrease in CL for drugs A and B, an increase in ${V}_{SS}$ for all drug types, and a slight increase in F for drug B. It is important to highlight that F remained below the unity in all simulations. This is expected given that all drug reabsorption events occur after the drug enters to the systemic circulation, therefore following the same mechanisms after IV or PO administration. Drug recirculating among the gut, gallbladder and the hepatocyte after PO administration does not contribute to the systemic exposure.

**Figure 6:**
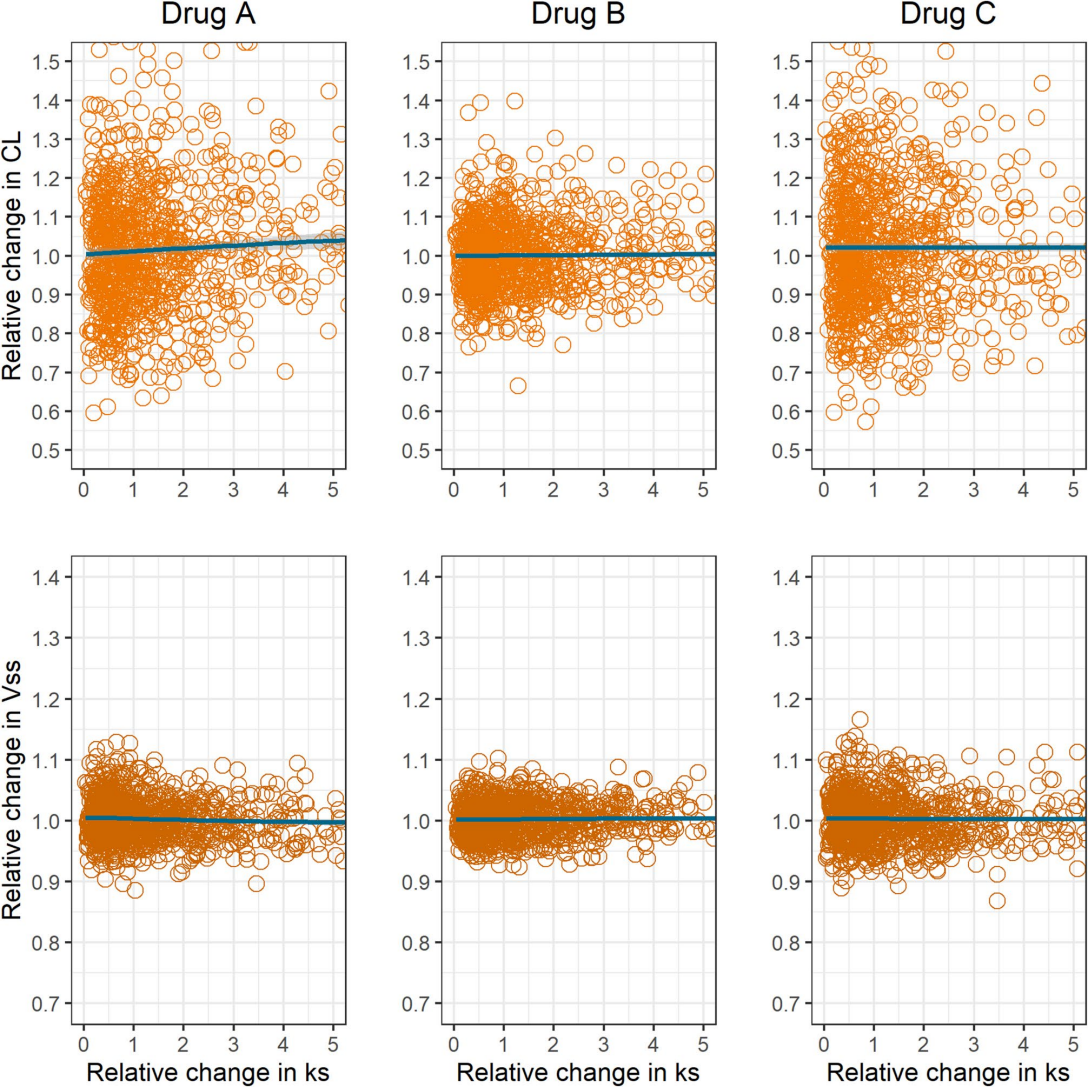
Sensitivity analysis with variability in all first-order rate constants, shown as scatterplots, for the mean impact of drug gastric secretion first-order rate constant (${k}_{s}$) on systemic clearance (CL) and volume of distribution at steady state (Vss).

Figure 6 shows the correlation between the relative change in the empirical systemic CL and ${V}_{SS}$ versus the relative change in the magnitude of ${k}_{s}$ obtained with the EGR model for the different drug types defined. Under this model, an increase in drug gastric secretion produces no significant mean change in the pharmacokinetic parameters, no matter the drug characteristics.

## Discussion

In this work, we have assumed two semi-mechanistic models for the representation of drug reabsorption according to different mechanisms of drug transference into the gastrointestinal lumen: hepatobiliary and gastric secretion. For both models, equations for CL, Vss and F were deduced and verified against empirical estimations obtained through simulation. Subsequently, the impact of drug reabsorption on the pharmacokinetic parameters of interest, considering variability for the whole system, was assessed by defining three drug types for which no limitations in intestinal permeability and minor intestinal elimination was assumed, therefore allowing the use of drug secretion (${k}_{hb}$ and ${k}_{s}$) as a surrogate of the extent of drug reabsorption.

As shown in sensitivity analyses for model EHR (Fig. 5), the increase in drug hepatobiliary secretion produces a reduction in the elimination clearance for drugs A and B while for drug C, in which renal excretion is the primary elimination route, the impact is marginal. The change in hepatic clearance for drugs A and B is explained by the competitive relationship between drug efflux and drug biotransformation and was captured in the model thanks to the representation of the liver as a peripheric compartment. In contrast, pharmacokinetic simulations performed with enterohepatic reabsorption models where the liver is included in the central compartment predicts no AUC alteration with changes in drug reabsorbed fraction, as it can be seen in a recent article by Okour and Brundage ^31^.

The magnitude of the alteration in drug clearance secondary to a change in hepatobiliary secretion depends on the relevance of hepatic clearance relative to other elimination routes and the hepatic extraction. Here, we observed a greater effect for drugs with low hepatic extraction (drug type A). For these drugs, a higher bypass effect of hepatobiliary secretion and drug reabsorption on hepatic clearance takes place due to the higher efficiency of drug absorption from the gut lumen into the systemic circulation. Valproic acid, a type A drug for which enterohepatic reabsorption has been reported ^14,32,33^, shows an increase in systemic clearance under the presence of several antibiotics ^34^. Mechanistically, this observation is commonly linked with antibiotic-mediated enterohepatic reabsorption interruption because of the reduction of bacteria β-glucuronidase in the gut lumen ^35^. We argue that glucuronidase mediated cleavage of valproic glucuronide might not be the major pathway for valproic acid reabsorption, given the low permeability the drug has at distal portions of the gut, where bacteria-mediated cleavage takes place. The increase in valproic acid clearance in the presence of antibiotics could be partially explained, within the proposed framework, by the reduction of valproic acid enteric reabsorption after competitive inhibition of MRP2 mediated hepatobiliary secretion. If this output is reduced, the higher intrahepatocyte concentrations will lead to a higher hepatic clearance. In a previous work we found a negative correlation between the systemic clearance and the reabsorbed fraction for this drug in healthy subjects ^26^. Padowski and Pollack also reported the importance of enterohepatic reabsorption in VPA disposition, particularly linked with MRP2 activity ^36^.

The volume of distribution was also affected by hepatobiliary secretion. For drugs B and C, the magnitude of change in Vss exceeded the corresponding change seen in CL. This is aligned with previous reports and our current understanding of the EHR impact on drug distribution. A clear negative correlation can be seen between volume of distribution and systemic clearance. As the drug bypasses its elimination in the liver and gets diverted to another peripheric compartment (gallbladder & afterwards gut lumen), the volume of distribution increases. It should be pointed out that the size of this effect depends on the extent of drug reabsorption relative to the extent to which that drug distributes into the peripheral tissues. The greater the distribution into the peripheral tissue, the smaller will be the effect of drug reabsorption on the estimated volume of distribution. The combined effect of drug reabsorption on clearance and volume of distribution), when present, will lead to an increase in drug half-life. Hepatobiliary drug secretion is governed by active transport and is subject to both intra and interindividual variability. Different individual characteristics and drug-drug interactions affecting efflux transporters activity can alter this process and subsequently drug clearance and volume of distribution.

Drug reabsorption had a smaller incidence on oral bioavailability as compared to its predicted effects on CL and Vss. As shown in Fig. 1, during the first passage of drug through the portal circulation after oral dosing, the amount extracted by the liver will be submitted to biotransformation, hepatobiliary secretion, or transportation back to the systemic circulation. The hepatobiliary secreted fraction will then have a second opportunity to be absorbed in the intestine and reach the systemic circulation. This was schematically illustrated in Table 2. An increase in the magnitude of this fraction therefore leads to a relative decrease in the extent of presystemic drug metabolism by reducing the time for drug exposure to the drug metabolizing enzymes. Recirculation gives repeated opportunities for drug absorption to the drug fraction extracted in the liver from the portal circulation. This effect is significant for drugs with high hepatic extraction (B), which show a basal reduced oral bioavailability since the biotransformation is very efficient during that first passage. For drugs with low hepatic extraction (A and C), the impact could be considered negligible because the basal oral bioavailability is close to 100%. Is important to point out that in all cases absolute bioavailability was relatively high given that presystemic elimination was the only considered limitation for drug absorption.

**Figure 7:**
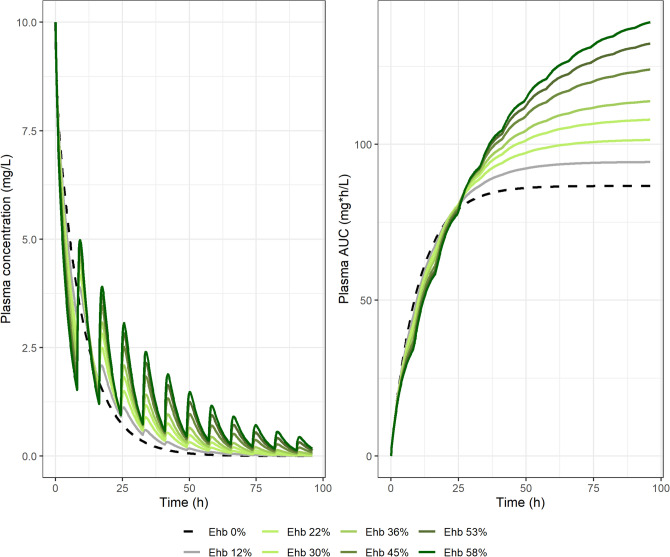
Plasma drug concentration (left) and AUC (right) versus time plotted for a type A drug changing the magnitude of hepatobiliary secretion first-order rate constant ($${k}_{hb})$$. $${E}_{hb}$$ corresponds to the hepatobiliary extraction defined as $${E}_{hb}= {k}_{hb}/\left({k}_{hc}+{{k}_{hb}+k}_{h}\right)$$.

The impact of enterohepatic reabsorption in plasma concentrations and AUC is further illustrated in Fig. 7 for a type A drug by plotting both plasma concentration versus time and partial AUC values versus time for different magnitudes of hepatobiliary secretion. This transference could be induced or inhibited by a perpetrator compound, altered by disease state, or affected by significant interindividual variability (sub-populations) with different mean reabsorbed fractions and therefore different systemic clearance. It can be observed that an increase in hepatobiliary secretion results in a significative increase in AUC (i.e. a reduction in systemic CL). Previous analyses reported by authors such as Shepard et al. in 1985 ^7^ and Okour et al. in 2019 ^31^, conclude that the extent of drug enterohepatic reabsorption has no impact in AUC. In a model-based analysis, the model structure has a strong impact in the outcome. What leads these authors to reach that conclusion is the inclusion of the hepatocyte in the central compartment, thereby ignoring the competition between the hepatobiliary secretion and drug metabolism occurring inside the hepatocyte. Therefore, their model did not account for the decrease in hepatic CL when drug reabsorption is augmented. With the model presented in this work, we make this competition explicit, and shed light into an important subject.

A scarcely studied scenario is where the drug is reabsorbed from the gastrointestinal tract after gastric (or eventually pancreatic) secretion. Figure 6 shows that for the drug types considered in the analysis, no impact of gastric secretion over systemic clearance and volume of distribution is predicted within the EGR model. The difference in relation to EHR is a function of the nature of drug secretion into the gastrointestinal lumen. While there is no competition between this transference and drug elimination, competition does exist with distribution processes. As this happens in the peripheral space, Vss is also not affected.

We previously analyzed the population pharmacokinetics of nevirapine, a weak base (pKa = 2.8) with high pH-dependent aqueous solubility, mainly eliminated through the liver with low hepatic extraction ^23^. Despite its negligible hepatobiliary transference, nevirapine shows secondary peaks after intravenous administration ^24^. A model for drug reabsorption describing nevirapine multiple peaks observed in plasma after oral administration was built. In this case, no correlation was observed between the random effects (interindividual variability) of drug clearance and fraction of reabsorbed drug, in accordance to what would be expected for a drug type A undergoing enterogastric reabsorption.

In a similar fashion, enteropancreatic reabsorption (EPR) could not be discarded for weak acids. Although the involvement of this process in drug recirculation has not been reported, pancreatic secretion and reabsorption it has been characterized for ions such as zinc ^37^. The pH-driven discrete pancreatic secretion of drugs through acinar cells is highly plausible and its discharge in the duodenum make drug reabsorption a likely scenario. Given the physiological background of the EPR process, conclusions similar conclusions to those obtained analyzing enterogastric reabsorption would be reached.

Under the hypothesis of the present work, in accordance with previous analyses reported for valproic acid and nevirapine, the correlation of random effects between drug reabsorption and systemic clearance estimated through a simpler and identifiable population pharmacokinetic model accounting for this process could give insight on the underlying mechanism. This is probably the most important contribution of this work: i.e., there are various potential mechanisms of drug reabsorption, each with their own corresponding pharmacokinetic impact.

Some limitations of this work should be considered. First-order kinetics were assumed in all drug transferences, although elimination and active efflux are saturable processes. A more complex scenario could be represented and further analyzed. This includes, for instance, drug auto-induction of the hepatobiliary secretion by increasing the expression of efflux transporters at the hepatobiliary barrier, which would lead to a hepatic clearance reduction and then to a non-linear kinetics scenario. We have previously hypothesized that this could be the case for phenytoin ^38^.

Another limitation of this analysis is that back conversion of metabolites in the gut leading to subsequent reabsorption of the parent drug was not explicitly accounted for in the models. This is a more complex scenario that could be further analyzed. An increase in this process will decrease the total (systemic) drug clearance, but hepatobiliary metabolite secretion would not affect the liver elimination capacity for the parent drug. Under the EHR model considered in this work, the kinetics of the sequential process involving: (i) hepatic metabolite formation, (ii) metabolite hepatobiliary secretion and (iii) back conversion in the gut; could be lumped into one apparent hepatobiliary transference first-order rate constant for the parent drug. Conclusions will not change, if metabolite formation (step i) is the increasing process, because a competitive relationship would exist between this reaction and other transferences occurring within the hepatocyte. Variability in steps (ii) and (iii) could alter the systemic clearance for the parent drug but not by affecting the hepatic clearance.

Overall, the work here presented clarifies the discussion on the impact of enterohepatic reabsorption on pharmacokinetic parameters, providing overarching conclusions that could be specifically analyzed for a specific drug from a systems perspective such as Physiologically Based Pharmacokinetic (PBPK) modeling. The simpler approach conducted in this work allows for the derivation and verification of theoretical equations for the different pharmacokinetic parameters in presence of a significant drug reabsorption process.

As expected, in accordance with other authors and current understanding, the volume of distribution can be increased with higher drug reabsorption. In addition, for drugs mainly eliminated at the liver, systemic clearance can be reduced with higher drug reabsorption. This is in line with the work of Yamaoka et al. ^9^, Horcovitz-Kovatz ^10^ and Peris-Ribera et al. ^8^. Furthermore, drug bioavailability could be increased by a higher drug enterohepatic reabsorption in drugs of high hepatic extraction. These findings can give background to explain and predict drug-drug interactions and other pharmacokinetic changes linked with hepatobiliary drug secretion, such as efflux transporter autoinduction or pathophysiological conditions. Finally, scarcely studied scenarios conducting to drug reabsorption were quantitatively assessed and compared with the most popular mechanism. To our knowledge, this is the first work addressing the dissimilar impact of drug reabsorption according to the underlying mechanism.

## Conclusion

The semi-mechanistic models implemented in this work gave a quantitative assessment on the impact of drug enteric secretion and drug reabsorption on primary pharmacokinetics parameters. In addition, the analysis showed that dissimilar pharmacokinetic repercussions can be expected depending on the underlying mechanism.

Our findings indicate that the magnitude of drug enterohepatic reabsorption is positively correlated with the volume of distribution, regardless of drug characteristics. Further, drug enterohepatic reabsorption can decrease the systemic clearance of drugs eliminated mainly through hepatic metabolism. In these cases, a greater impact is expected for drugs showing low hepatic extraction. For renally cleared drugs, no significant impact is expected. Finally, the oral bioavailability of drugs with high liver extraction could result increased following an increase in the extent of reabsorbed drug.

Conversely, when drug reabsorption takes place after gastric secretion the magnitude of enterogastric reabsorption will have a negligible impact on pharmacokinetic parameters.

This work provides background to explain and forecast the role that these processes can play in pharmacokinetic variability, including drug-drug interactions and disease states.

## Supplementary Information

Supplementary Information.
