# Supplementary material for: Enteric reabsorption processes and their impact on drug pharmacokinetics
Source: Sci Rep. 2021 Mar 11;11:5794. doi: 10.1038/s41598-021-85174-w (PMC7952424; doi:10.1038/s41598-021-85174-w)
Supplement: Supplementary file 1 — Supplementary Information. [file 41598_2021_85174_MOESM1_ESM.pdf]

**Enteric reabsorption: influence on drug pharmacokinetics depending on the underlying mechanism**

Manuel Ibarra*[1], Iñaki F. Trocóniz[2,3] and Pietro Fagiolino[1]

1. Department of Pharmaceutical Sciences. Faculty of Chemistry. *Universidad de la República*. Montevideo, Uruguay. 2. Pharmacometrics and Systems Pharmacology Research Unit, Department of Pharmaceutical Technology and Chemistry, School of Pharmacy and Nutrition, University of Navarra. Pamplona, Spain. 3. IdiSNA; Navarra Institute for Health Research, Pamplona, Spain.

## SUPPLEMENTARY INFORMATION

### 1. TABLES

Table Ia – Parameters implemented for the sensitivity analysis in the EHR model. Included as: mean values (standard deviation). A log-normal distribution was assumed for all rate constants. The volume of distribution for the central compartment was set to 10 L.

| Drug | $k_h$ $(h^{-1})$ | $k_g$ $(h^{-1})$ | $k_r$ $(h^{-1})$ | $k_a$ $(h^{-1})$ | $k_{ch}$ $(h^{-1})$ | $k_{hc}$ $(h^{-1})$ | $k_{cg}$ $(h^{-1})$ | $k_{gh}$ $(h^{-1})$ | $k_{hb}$ $(h^{-1})$ |
|---|---|---|---|---|---|---|---|---|---|
| **A** | 1.1 (0.2) | 0.06 (0.2) | 0.01 (0.2) | 1.5 (0.3) | 0.3 (0.1) | 2.5 (0.1) | 0.2 (0.1) | 0.2 (0.1) | 1.0 (1.0) |
| **B** | 7.0 (0.2) | 0.2 (0.2) | 0.07 (0.2) | 1.5 (0.3) | 1.0 (0.1) | 1.5 (0.1) | 0.2 (0.1) | 1.0 (0.1) | 3.0 (1.0) |
| **C** | 0.35 (0.2) | 0.0 | 0.4 (0.2) | 1.5 (0.3) | 1.0 (0.1) | 2.5 (0.1) | 0.2 (0.1) | 0.5 (0.1) | 1.0 (1.0) |

Table Ib – Parameters implemented for the sensitivity analysis in the EGR model. Included as: mean values (standard deviation). A log-normal distribution was assumed for all rate constants. The volume of distribution for the central compartment was set to 10 L.

| Drug | $k_h$ $(h^{-1})$ | $k_g$ $(h^{-1})$ | $k_r$ $(h^{-1})$ | $k_a$ $(h^{-1})$ | $k_{ch}$ $(h^{-1})$ | $k_{hc}$ $(h^{-1})$ | $k_{cg}$ $(h^{-1})$ | $k_{gh}$ $(h^{-1})$ | $k_s$ $(h^{-1})$ | $k_{sc}$ $(h^{-1})$ | $k_{cs}$ $(h^{-1})$ | $k_{sh}$ $(h^{-1})$ |
|---|---|---|---|---|---|---|---|---|---|---|---|---|
| **A** | 1.0 (0.2) | 0.25 (0.2) | 0.03 (0.2) | 1.5 (0.3) | 1.0 (0.1) | 2.5 (0.1) | 0.2 (0.1) | 0.1 (0.1) | 0.5 (1.0) | 0.5 (0.1) | 0.15 (0.1) | 0.1 (0.1) |
| **B** | 5.0 (0.2) | 0.5 (0.2) | 0.15 (0.2) | 1.5 (0.3) | 1.0 (0.1) | 1.5 (0.1) | 0.2 (0.1) | 1.0 (0.1) | 0.5 (1.0) | 0.5 (0.1) | 1.5 (0.1) | 0.1 (0.1) |
| **C** | 0.3 (0.2) | 0.0 | 0.4 (0.2) | 1.5 (0.3) | 1.0 (0.1) | 2.5 (0.1) | 0.2 (0.1) | 0.1 (0.1) | 0.5 (1.0) | 0.5 (0.1) | 0.15 (0.1) | 0.1 (0.1) |

## 2. R-SCRIPTS AND MLXTRAN CODE TO IMPLEMENT SIMULATIONS FOR MODELS EHR AND EGR AFTER ORAL AND INTRAVENOUS ADMINISTRATION

**R-script enterohepatic reabsorption model**

```
### Enterohepatic reabsorption model (EHR) ###

library(mlxR)

# Defining parameter values for the following constant rates (h-1)

k41 <- 1.5      # absorption & reabsorption from the gastrointestinal compartment

k40 <- 0.06     # intestinal elimination

k42 <- 0.2      # hepatic uptake of drug coming from the gastrointestinal compartment through
portal venous blood

k12 <- 0.3      # hepatic drug uptake from systemic circulation

k14 <- 0.2      # enterocyte uptake from systemic circulation

k10 <- 0.01     # renal elimination

k21 <- 2.5      # liver-to-central transference

k20 <- 1.1      # hepatic elimination

k23 <- 1       # hepatobiliary secretion

Bb <- 50       # gallbladder release


# Defining value for the volume of distribution of the central compartment (L)

V1 <- 10

# Steps for gallbladder release

d1 <- 8

d2 <- 0.2

d3 <- d1+d2

step1 <- list(time=seq(d1,300,by=d3), amount=1, adm=1)

step2 <- list(time=seq(d3,300,by=d3), amount=-1, adm=1)

# Defining treatment

dose  <- list(time=0, amount=100, adm=2)

# Parameter vector for inclusion in simulx

p <- c(ka = k41,

       kel_g = k40,

       kg_l = k42,
```

```
    kc_l = k12,
    kc_g = k14,
    kel_r = k10,
    kl_c = k21,
    kel_l = k20,
    khb = k23,
    B = Bb,
    Vc = V1
)
# Output definition
f <- list(name = c('Ac','Cc','Agb','Aliv','Agut','AUCgut','Fr','AUC','Eren','Eliv','Egut','Relgb'),
      time=seq(0,96,by=0.1))
# Performing simulation for intravenous bolus administration
IV_EHC <- simulx(model= "Supp_EHCmodel_IV.txt",
          treatment= list(step1, step2, dose),
          parameter = p,
          output = f)
# Ploting
A <- cbind(IV_EHC$Ac, IV_EHC$Agb['Agb'], IV_EHC$Aliv['Aliv'],IV_EHC$Agut['Agut'])
D <- melt(A, id="time", value.name = "amount", variable.name = "compartment")
ggplot(data=res$Relgb) + geom_line(aes(x=time, y=Relgb), size=1, color="red") # Gallbladder release pattern
ggplot(D, aes(time, amount,colour=compartment)) + geom_line() +
  facet_wrap(~compartment,scales="free") +
  labs(x="Time (h)", y="Amount (mg)")                    # Amounts vs time

# Performing simulation for oral administration
PO_EHC <- simulx(model= "Supp_EHCmodel_PO.txt",
            treatment= list(step1, step2, dose),
            parameter = p,
            output = fo)
```

**Mlxtran code for intravenous administration into the EHR model (Supp_EHCmodel_IV.txt)**

```
[LONGITUDINAL]

input= {ka, kel_g, kg_l, kc_l, kl_c, kel_l, khb, kc_g, kel_r, B, Vc}


PK:

compartment(cmt=1, amount=Relgb)

compartment(cmt=2, amount=Ac)


iv(adm=1, cmt=1)

iv(adm=2, cmt=2)


EQUATION:

odeType = stiff

t0 = 0

Relgb_0 = 0

ddt_Relgb=0


ddt_Agut = Relgb*B*Agb + kc_g*Ac - (ka+kg_l+kel_g)*Agut

ddt_Ac = ka*Agut  +kl_c*Aliv - (kc_l+kel_r+kc_g)*Ac

ddt_Aliv = kg_l*Agut + kc_l*Ac - (kel_l+kl_c+khb)*Aliv

ddt_Agb = khb*Aliv - Relgb*B*Agb


ddt_Eren = kel_r*Ac

ddt_Egut = kel_g*Agut

ddt_Eliv = kel_l*Aliv

ddt_Fr   = ka*Agut


Cc = Ac/Vc

ddt_AUCgut = Agut

ddt_AUC = Cc
```

**Mlxtran code for oral administration into the EHR model**

**(Supp_EHCmodel_PO.txt)**

```
[LONGITUDINAL]
input= {ka, kel_g, kg_l, kc_l, kl_c, kel_l, khb, kc_g, kel_r, B, Vc}

PK:
compartment(cmt=1, amount=Relgb)
compartment(cmt=2, amount=Ac)
compartment(cmt=3, amount = Agut)

iv(adm=1, cmt=1)
iv(adm=2, cmt=3)

EQUATION:
odeType = stiff
t0 = 0
Relgb_0 = 0
ddt_Relgb=0

ddt_Agut = Relgb*B*Agb + kc_g*Ac - (ka+kg_l+kel_g)*Agut
ddt_Ac = ka*Agut  +kl_c*Aliv - (kc_l+kel_r+kc_g)*Ac
ddt_Aliv = kg_l*Agut + kc_l*Ac - (kel_l+kl_c+khb)*Aliv
ddt_Agb = khb*Aliv - Relgb*B*Agb

ddt_Eren = kel_r*Ac
ddt_Egut = kel_g*Agut
ddt_Eliv = kel_l*Aliv

Cc = Ac/Vc
```

ddt_AUCgut = Agut

ddt_AUC = Cc

**R-script enterogastric reabsorption model**

```
### Enterogastric reabsorption model (EGR) ###

library(mlxR)


# Defining parameter values for the following constant rates (h-1)


k51 <- 1.5      # absorption & reabsorption from the gastrointestinal compartment

k50 <- 0.25     # intestinal elimination

k52 <- 0.1      # hepatic uptake of drug coming from the gastrointestinal compartment through portal venous blood

k12 <- 1        # hepatic drug uptake from systemic circulation
```

```
k15 <- 0.2      # enterocyte uptake from systemic circulation

k10 <- 0.03     # renal elimination

k21 <- 2.5      # liver-to-central transference

k20 <- 1        # hepatic elimination

k23 <- 1        # hepatobiliary secretion

k13 <- 0.15     # parietal cell uptake from cental compartment

k31 <- 0.5      # parietal cell-to-central transference

k34 <- 0.5      # secretion of drug into the gastric lumen

k32 <- 0.1      # hepatic uptake from arterial irrigation

Bb <- 50        # stomach release


# Defining value for the volume of distribution of the central compartment (L)


V1 <- 10


# Steps for stomach release


d1 <- 8

d2 <- 0.2

d3 <- d1+d2

step1 <- list(time=seq(d1,50,by=d3), amount=1, adm=1)

step2 <- list(time=seq(d3,50,by=d3), amount=-1, adm=1)


# Defining treatment

dose  <- list(time=0, amount=100, adm=2)


# Parameter vector for inclusion in simulx


p <- c(ka = k51,

     kgut = k50,
```

```
    kg_l = k52,
    kc_l = k12,
    kc_g = k15,
    kr = k10,
    kl_c = k21,
    kliv = k20,
    kc_s = k13,
    ks_c = k31,
    ks_l = k32,
    ks = k34,
    B = Bb,
    Vc = V1
)

# Output definition

f <- list(name = c('Ac', 'As','Aliv','Ajg','Agut','AUCgut','AUC','Eren','Eliv','Egut','Relgb'),
      time=seq(0,50,by=0.1))


# Performing simulation for intravenous bolus administration

IV_EGC <- simulx(model= "Supp_EGCmodel_IV.txt",
         treatment= list(step1, step2, dose),
         parameter = p,
         output = f)

# Ploting

A <- cbind(res$Ac, res$As['As'], res$Aliv['Aliv'],res$Agut['Agut'],res$Ajg['Ajg'])
```

```
D <- melt(A, id="time", value.name = "amount", variable.name = "compartment")

ggplot(data=res$Relst) + geom_line(aes(x=time, y=Relst), size=1, color="red") # Stomach release pattern

ggplot(D, aes(time, amount,colour=compartment)) + geom_line() +
  facet_wrap(~compartment,scales="free") +
  labs(x="Time (h)", y="Amount (mg)")                          # Amounts vs time


# Performing simulation for oral administration

PO_EGC <- simulx(model= "Supp_EGCmodel_PO.txt",
                 treatment= list(step1, step2, dose),
                 parameter = p,
                 output = fo)
```

**Mlxtran code for intravenous administration into the EHR model**

**(Supp_EGCmodel_IV.txt)**

```
[LONGITUDINAL]
input= {ka, kgut, kg_l, kl_c, kliv, kc_l,kc_g,kc_s,ks_c,ks_l, ks, B, Vc, kr}


PK:
compartment(cmt=1, amount=Relst)
compartment(cmt=2, amount=Ac)

iv(adm=1, cmt=1)
iv(adm=2, cmt=2)
```

```
EQUATION:
odeType = stiff
t0 = 0
Relgb_0 = 0
ddt_Relst=0

ddt_Agut = -(ka+kgut+kg_l)*Agut + Relst*B*Ajg + kc_g*Ac
ddt_Ac = ka*Agut+kl_c*Aliv+ks_c*As-(kc_l+kc_g+kc_s+kr)*Ac
ddt_Aliv = kc_l*Ac +ks_l*As - (kl_c+kliv)*Aliv + kg_l*Agut
ddt_As = kc_s*Ac - (ks_c+ks_l+ks)*As
ddt_Ajg = ks*As- Relst*B*Ajg

Cc = Ac / Vc
ddt_AUCgut = Agut
ddt_AUC = Cc
ddt_Eren = kr*Ac
ddt_Egut = kgut*Agut
ddt_Eliv = kliv*Aliv
```

**Mlxtran code for oral administration into the EHR model**

**(Supp_EGCmodel_PO.txt)**

```
[LONGITUDINAL]
input= {ka, kgut, kg_l, kl_c, kliv, kc_l,kc_g,kc_s,ks_c,ks_l, ks, B, Vc, kr}



PK:
compartment(cmt=1, amount=Relst)
compartment(cmt=2, amount=Ac)
compartment(cmt=3, amount = Agut)
```

```
iv(adm=1, cmt=1)

iv(adm=2, cmt=3)


EQUATION:

odeType = stiff

t0 = 0

Relst_0 = 0

ddt_Relst=0


ddt_Agut = -(ka+kgut+kg_l)*Agut + Relst*B*Ajg + kc_g*Ac

ddt_Ac = ka*Agut+kl_c*Aliv+ks_c*As-(kc_l+kc_g+kc_s+kr)*Ac

ddt_Aliv = kc_l*Ac +ks_l*As - (kl_c+kliv)*Aliv + kg_l*Agut

ddt_As = kc_s*Ac - (ks_c+ks_l+ks)*As

ddt_Ajg = ks*As- Relst*B*Ajg


Cc = Ac / Vc

ddt_AUCgut = Agut

ddt_AUC = Cc

ddt_Eren = kr*Ac

ddt_Egut = kgut*Agut

ddt_Eliv = kliv*Aliv
```
